# Comprehensive Analyses of miRNAs Revealed miR-92b-3p, miR-182-5p and miR-183-5p as Potential Novel Biomarkers in Melanoma-Derived Extracellular Vesicles

**DOI:** 10.3389/fonc.2022.935816

**Published:** 2022-07-08

**Authors:** Dennis Gerloff, Stefanie Kewitz-Hempel, Gerd Hause, Jovine Ehrenreich, Linda Golle, Tim Kingreen, Cord Sunderkötter

**Affiliations:** ^1^ Department of Dermatology and Venereology, University Hospital Halle (Saale), Martin-Luther-University Halle-Wittenberg, Halle (Saale), Germany; ^2^ Biocenter, Martin Luther University Halle-Wittenberg, Halle, Germany

**Keywords:** melanoma, extracellular vesicles (EVs), miRNAs, biomarker, skin cancer (melanoma)

## Abstract

Extracellular vesicles (EVs) are important mediators in the intercellular communication, influencing the function and phenotype of different cell types within the tumor micro-milieu and thus promote tumor progression. Since EVs safely transport packages of proteins, lipids and also nucleic acids such as miRNAs, EVs and their cargo can serve as diagnostic and prognostic markers. Therefore, the aim of this study was to investigate EV embedded miRNAs specific for melanoma, which could serve as potential biomarkers. In contrast to previous studies, we not only analysed miRNAs from EVs, but also included the miRNA profiles from the EV-secreting cells to identify candidates as suitable biomarkers. While the characterization of EVs derived from normal melanocytes and melanoma cells showed largely comparable properties with regard to size distribution and expression of protein markers, the NGS analyses yielded marked differences for several miRNAs. While miRNA load of EVs derived from normal human epidermal melanocytes (NHEMs) and melanoma cells were very similar, they were highly different from their secreting cells. By comprehensive analyses, six miRNAs were identified to be enriched in both melanoma cells and melanoma cell-derived EVs. Of those, the accumulation of miR-92b-3p, miR-182-5p and miR-183-5p in EVs could be validated *in vitro*. By functional network generation and pathway enrichment analysis we revealed an association with different tumor entities and signaling pathways contributing melanoma progression. Furthermore, we found that miR-92b-3p, miR-182-5p and miR-183-5p were also enriched in EVs derived from serum of melanoma patients. Our results support the hypothesis that miRNAs derived from EVs can serve as prognostic or diagnostic liquid biopsy markers in melanoma. We identified EV-derived miRNAs and showed that those miRNAs, which were enriched in melanoma cells and EVs, are also found elevated in serum-derived EVs of patients with metastatic melanoma, but not in healthy subjects.

## Introduction

Melanoma is one of the most aggressive malignant tumors worldwide with a still increasing incidence. The endogenously controlled processes that drive tumor growth and cancer progression have long been studied. A second important aspect is the influence of cell-cell communication within the tumor microenvironment. Here intercellular crosstalk occurs direct by cell-to-cell contact *via* adhesion molecules or electrical coupling, and indirectly through classical signaling *via* cytokines, growth factors, and extracellular vesicles (EVs). The latter are attracting increasing interest as potent mediators of intercellular signaling, since they protect proteins, lipids and especially nucleic acids against degradation and because they present potential prognostic and diagnostic biomarkers.

EVs are lipid-membrane bound, cell-derived nanoparticles secreted by all cells under physiological and pathological conditions. They are distinguished by size and biogenesis into exosomes, microvesicles and apoptotic bodies (1, 2).

Exosomes are very small (50 – 150 nm) lipid vesicles derived from the endosomal compartment. They are formed in multivesicular bodies (MVB) and released in the extracellular space by fusion with the cell membrane (1–4). They transport lipids (e.g. cholesterol), proteins (e.g. ALIX, HSP70), DNA as well as coding and non-coding RNAs (e.g. miRNAs) (2, 4).

Microvesicles are larger in size (100 – 1000 nm) than exosomes. They are membrane-derived vesicles released in the extracellular environment by budding (shedding) or fission of the plasma membrane (1, 2, 4, 5). Similar to exosomes, microvesicles are loaded with lipids, proteins and nucleic acids (2, 4).

Apoptotic bodies are the largest extracellular vesicles (1000 - 2000 nm). They are released during the disassembly of dying cells. Because of their size apoptotic bodies contain larger amounts of lipids, proteins and nucleic acids, but also cellular components such as parts of cytosol, micronuclei or intact organelles. In contrast to microvesicles and exosomes, apoptotic bodies are not known to mediate intercellular communication, but are incorporated and degraded by phagocytic cells (e.g. macrophages) (1, 2, 4, 5).

The updated guideline of the international society for extracellular vesicles states that i) EVs should now be defined as natural particles with a lipid double layer secreted by cells and unable to replicate (not containing a nucleus) (6); ii) they should be differentiated according to size, density, biochemical content and surface markers. Exosomes and small microvesicles (100 - 200 nm) are also often referred to as small EVs *(*50 - 200 nm), because they both express typical surface markers such as tetraspanins CD63, CD81 and CD9 (1, 2, 4, 5), which distinguish them from apoptotic bodies.

One of the important distinct function of EVs is the transport of microRNAs (miRNAs), since in contrast to lipids and proteins most RNAs are rapidly degraded in the extracellular space. Protected by EVs, miRNAs are able to impact cell-cell communication in a way which has long been neglected.

MicroRNAs are small (~22 nt) single stranded non-coding RNAs, which post-transcriptionally regulate protein expression by interacting with target mRNAs. They are transcribed by RNA polymerase II and processed in several steps (7). The mature miRNA single strand is incorporated into the RNA-induced silencing complex (RISC) and functions as guide for binding to the complementary seed regions in the 3’ untranslated region (3’UTR) of target mRNAs (7, 8). The binding of RISC to the 3’UTR inhibits protein translation by the repression of translational initiation process and ribosome assembly as well as degradation of mRNA (7).

Posttranslational regulations by miRNAs are involved in physiological processes as embryogenesis, differentiation and development, but also in pathological events as cancer development and other diseases (7).

We and others have provided evidence that cell-cell communication within the tumor microenvironment and e.g. modifying macrophages is partially mediated by miRNAs transported in EVs (2, 9–13). Some of these miRNAs modify cancer cells, by e.g. mediating drug resistance (12, 14–16) or promoting metastasis (15, 17) in different cancer entities (e.g. breast cancer, glioma, small cell lung cancer and cervical squamous cell carcinoma).

miR-155 from melanoma-derived EVs induces matrix reprogramming and promotes angiogenesis by inducing the activation of carcinoma-associated fibroblasts (CAFs) (18). We revealed that the exosomal miR-125b-5p secreted by melanoma cells induces pro-inflammatory tumor-associated macrophages (TAMs) by targeting LIPA, resulting in increased M1 phenotype marker expression, e.g. IL-1β, CCL1, CCL2 (9).

Since their secretion is increased in malignancies and the miRNA cargo differ between normal and cancer cells, miRNAs embedded in EVs are intended to be used as liquid biobsy markers in cancer (19–22).

Therefore, the aim of this study was to investigate EV embedded miRNAs specific for melanoma, which could serve as potential biomarkers. In contrast to previous studies, we not only analysed miRNAs cargo of EVs, but also included the miRNA profiles from the EV-secreting cells to identify candidates as suitable biomarkers.

We revealed six miRNAs that were significantly enriched in EVs from melanoma cell lines and in the corresponding cells. Functional Network analyses predicted an association of these miRNAs in various cancer entities and tumor-promoting signaling pathways.

The *in vitro* enrichment in EVs could be validated, in EVs derived from patient samples, for the three miRNAs: miR-92b-3p, miR-182-5p and miR183-5p. Therefore, these EV loaded miRNAs may serve as potential novel prognostic or diagnostic liquid biopsy markers in melanoma.

## Materials and Methods

### Cell Cultures

Melanoma cell lines (WM9, WM902B, WM35, BLM, MV3 and A375) were cultured in DMEM, supplemented with 10% fetal calve serum (FCS) (Sigma Aldrich, Taufkirchen, Germany) and 1% penicillin-streptomycin (Sigma Aldrich, Taufkirchen, Germany). Cell lines, used in the NGS analyses, represent melanoma in different progression stages (WM35 radial growth phase (RGP), WM902B vertical growth phase (VGP) and WM9 metastatic melanoma). Melanoma cell lines were provided from the Department of Dermatology, University of Münster, Germany. Primary normal human epidermal melanocytes (NHEM) were isolated in our laboratory from juvenile foreskins and cultured in medium 254 (Thermo Fisher Scientific, Waltham, Massachusetts, USA) including human melanocyte growth supplement (HMGS) and 1% penicillin-streptomycin. All cells were incubated at 37°C and 5% CO_2_.

### Isolation and Analysis of Small Extracellular Vesicles

EVs were isolated and characterized according to the 2018 consensus statement on minimal information for studies of extracellular vesicles (MISEV2018) (6). Cells were cultured for 48 h in DMEM supplemented with 10% exosome depleted FCS (Thermo Fisher Scientific, Waltham, Massachusetts, USA) and 1% penicillin-streptomycin. Supernatants (30 ml) were collected and centrifuged for 10 min at 300 g to remove cells and cell debris, followed by 30 min at 10000 g to remove larger vesicles. Afterwards the supernatants were filtered through a 0.2 µm filter and centrifuged at 100000 g for 1.5 h. Pelleted EVs were washed with PBS and centrifuged for another 1.5 h at 100000 g. Centrifugation was performed using a Sorvall WX+ Ultra Centrifuge, with SureSpin 632 rotor (k-factor 194) (Thermo Fisher Scientific, Waltham, Massachusetts, USA). EVs were resuspended in PBS. EV analysis was performed by nanoparticle tracking analysis (NTA) using a NanoSight NS300 (Malvern Panalytical, Kassel, Germany). Therefore, EVs were isolated or samples were diluted (conditioned media (1:100) or patient serum (1:1000)) without isolation and analysed from three independent biological samples. Measurements were performed at a controlled temperature of 22°C. For each sample, three measurements of 30 s were performed. EV concentration and size was calculated by the NanoSight software.

### Isolation of Human Serum-Derived EVs

For the initial testing of miRNA enrichment in serum-derived EVs, melanoma patients were included without special criterias. Serum of melanoma patients (8) and healthy donors (8) was collected at the Department of Dermatology and Venereology (University Hospital Halle (Saale), Germany). Patient characteristics are summarized in **Supplementary Table 1**. All patients were bearing advanced metastatic melanoma (stage IIIC – IV) while healthy donors had no known malignancies or atypical moles, at the timepoint when samples were collected. To remove cells and cell debris the collected sera were centrifuged at 300 g for 10 min, followed by another centrifugation step at 10000 g for 30 min. Then EVs were isolated from 100 µl serum by size exclusion chromatography (sec) using Exo-spin colums according manufactures instructions (Cell Guidance Systems, Cambridge, UK). Serum derived EVs were analyzed by western blot and NTA (**Figure S1**). The study was conducted in accordance with good clinical practice guidelines and the declaration of Helsinki. All patients gave their written informed consent. The ethical committee of the medical faculty of the Martin-Luther-University Halle-Wittenberg approved the study.

### Transmission Electron Microscopy

To prepare TEM-samples 3 µl of the dispersion were spread onto Cu-grids coated with a formvarfilm. After 1 min of adsorption, excess liquid was blotted off with filter paper. Subsequently the grids were air-dried for 15 sec, washed with water (3 times for 1 min), placed on a droplet of 2% aqueous uranyl acetate and drained off after 1 min. The dried specimens were examined with an EM 900 transmission electron microscope (Carl Zeiss Microscopy, Jena, Germany) at an acceleration voltage of 80 kV. Electron micrographs were taken with a Variospeed SSCCD camera SM-1k-120 (TRS, Moorenweis, Germany).

### Immunoblot Analyses

Cells and EVs were lysed by RIPA buffer for 30 min at 4°C. 20 µg of protein extracts were resolved by SDS–PAGE and blotted to nitrocellulose membranes and probed with the following antibodies: anti-CD81 (5A6) (1:200), anti-CD63 (MX-49.129.5) (1:500), anti-HSP70 (3A3) (1:500), anti-ALIX (1A4) (1:250), anti-CANX (AF18) (1:500) (Santa Cruz, Dallas, USA) and anti-CD9 (CGS12A) (1:1000) (Cell Guidance Systems, Cambridge, UK). Antibody incubation was performed in 5% milk at 4°C over night. For antibody detection, blots were incubated for 1 h at room temperature with m-IgGκ BP-HRP (1:5000) (Santa Cruz, Dallas, USA) or anti-mouse IgG-HRP (1:2000) (Cell Signaling Technology, Leiden, Netherlands). Chemiluminescent detection was performed using Amersham ECL Prime (GE Healthcare, Amersham, UK).

### RNA Isolation and Analyses

Total RNA was extracted from cells or extracellular vesicles using TriFast™ reagent (Peqlab, Erlangen, Germany), according manufacturer’s protocol. RNA quality and quantity was analysed by Agilent bioanalyser (Agilent, Santa Clara, California, USA). MiRNA quantification was performed by qRT-PCR using TaqMan^®^ MicroRNA Reverse Transcription Kit and TaqMan^®^ Universal Master Mix II according manufacturer’s instructions (Thermo Fisher Scientic). Values were normalized by RNUB6 for cells, while for extracellular vesicles values were normalized by miR-16, because it was highly and stably expressed in our NGS analysis and it was earlier reported as endogenous normalization miRNA in exosomes (23). Relative fold changes were calculated by 2^-ΔΔCt^ method (24), comparing the values to the mean of the control group. MiRNA assays were purchased from Thermo Fisher Scientific (Thermo Fisher Scientific, Waltham, Massachusetts, USA).

### Small RNA-Seq

10 ng (EVs) or 50 ng (cells) of total RNA was used in the small RNA protocol with the NEXTflex Small RNA-seq Kit v3 (Bioo Scientific) according to the instructions of the manufacturer. A pool of libraries was used for sequencing at a concentration of 10 nM. Sequencing of 1x75 bp was performed with an Illumina NextSeq 550 sequencer at the sequencing core facility of the IZKF Leipzig (Faculty of Medicine, University Leipzig) according to the instructions of the manufacturer. Demultiplexing of raw reads, adapter trimming and quality filtering was done according to Stokowy et al. (25), using the adapter sequences of the NEXTflex kit containing random bases next to the library insert. Mapping against the human reference genome (hg38) and miRbase reference sequences (v22) was done using Bowtie2 (26). Read counts were calculated with the R bioconductor package Rsamtools (http://bioconductor.org/packages/release/bioc/html/Rsamtools.html) and normalised using the DESeq2 (27) and EdgeR (28) R bioconductor packages.

### Heatmaps and Statistics

For the statistical analyses and graphical representation, Qlucore Omics Explorer and Graph Pad Prism software was used. Venn diagram was created using FunRich software (29). Differential expression fold-changes and p-values of the NGS data were calculated by Qlucore Omics Explorer. Significant altered miRNAs with p ≤ 0.05 were selected and Benjamini-Hochberg adjustment was used to adjust p-values. Adjusted p-values ≤ 0.05 were considered significant. To prove the statistical significands of the data, two tailed Student’s t-test or Mann-Whitney U-test was performed, depending on Gaussian distribution, which was evaluated by the Levene test. A p-value ≤ 0.05 was considered as statistical significant.

### miRNA Pathway Analysis

For computational miRNA pathway analysis we used MIENTURNET (MIcroRNAENrichmentTURnedNETwork) (30). Pathway analyses were performed for miR-92b-3p, miR-125b-5p, miR-182-5p, miR-183-5p and miR-221-3p by the default settings, using KEGG and Reactome. For miRNA-target network analysis, targets validated as strong based on miRTarBase were used.

## Results

### Isolation and Characterization of Small Extracellular Vesicles Derived From NHEMs and Melanoma Cells

**Figure 1:**
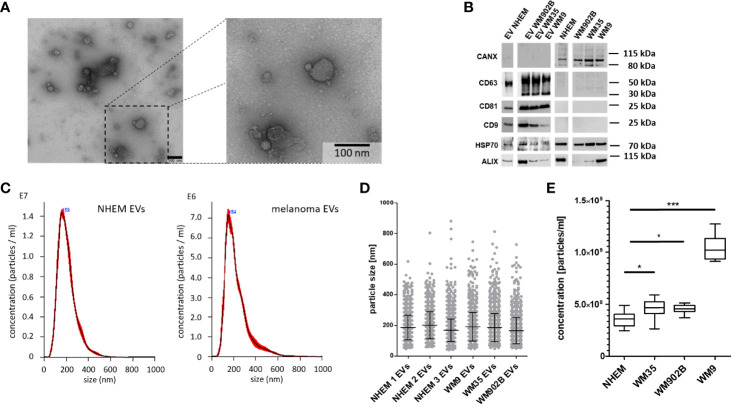
Isolation and characterization of extracellular vesicles. **(A)** Representative image of transmission electron microscopy (TEM) analyses of isolated EVs from melanoma cell line conditioned medium. Scale bar represents 100 nm. **(B)** Western blot analyses for EVs and corresponding cells investigated CD63, CD81, CD9, HSP70, ALIX and CANX. **(C)** Exemplary nanoparticle tracking analyses (NTA) for EVs derived from NHEMs and melanoma cells. **(D)** NTA showed a similar size distribution of EVs derived from NHEMs and melanoma cells. **(E)** NTA revealed that melanoma cells release higher concentrations of EVs. Graph represents EV concentrations of at least three biological independent experiments (*p ≤ 0.05; ***p ≤ 0.001).

In order to reveal the peculiarities of tumor-derived EVs, comparative analyses of miRNAs loaded into EVs from normal melanocytes and melanoma cells as well as the endogenous miRNA expression inside these cells were performed. For this purpose, primary normal human epidermal melanocytes (NHEM) from three different donors and three melanoma cell lines (WM35, WM9 and WM902B) were briefly cultured (48 h). EVs were isolated and characterized according to the 2018 consensus statement on minimal information for studies of extracellular vesicles (MISEV2018) (6). The separation of EVs from conditioned medium (CM) was performed by different centrifugation steps, including ultracentrifugation as previously described (9, 31). TEM confirmed the size distribution of the isolated EVs (~ 100 nm) and characteristic ultrastructure (**Figure 1A**). In western blot analyses an accumulation of the EV surface markers CD9, CD81 and CD63 as well as the cytosolic proteins HSP70 and ALIX were found in the lysates of EVs (6). In contrast, no accumulation in the EV fraction was found for Calnexin (CANX). Since CANX originates from the endoplasmic reticulum, enrichment would indicate cellular contamination of the EV fraction (**Figure 1B**). Nanoparticle tracking analysis (NTA) showed a similar size distribution of isolated EVs (± 150 nm) derived from NHEMs or melanoma cell lines (**Figures 1C, D**). To analyse the quantity of EVs derived from melanoma cells or melanocytes, cells were incubated for 24 h and EVs were measured by NTA. Melanoma cell-derived EVs were significantly increased compared to the EVs from normal melanocytes. The supernatant of the metastatic cell line WM9 showed the highest concentration of EVs (**Figure 1E**).

EVs derived from normal melanocytes and melanoma cells show largely comparable properties with regard to size distribution and surface marker expression.

### NGS Analysis of miRNA Cargo in EVs and miRNA Profiles of Corresponding Cells

**Figure 2:**
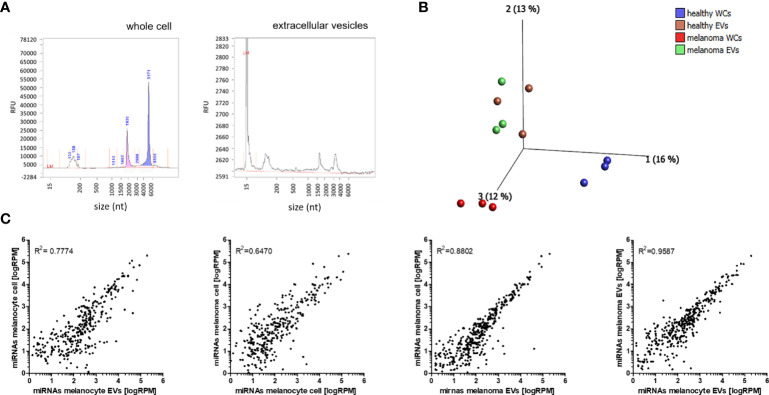
NGS analyses of miRNA cargo in EVs and miRNA profiles of corresponding cells. **(A)** Representative Bioanalyser quality control of RNA derived from whole cells and EVs. **(B)** Principle component analysis (PCA) of the miRNA profiles of NHEMs, melanoma cells and the corresponding EVs. (WCs: whole cells; EVs: extracellular vesicles) **(C)** Scatter plots showing the correlation of miRNA profiles between the average expressions of three biological replicates of cells and EVs.

To investigate the miRNA cargo in EVs and the miRNA expression in the corresponding cells, total RNA was isolated and analysed by Bioanalyser system (**Figure 2A**). While the cell-derived RNA shows a high enrichment for rRNAs, EV isolated RNA shows no rRNA, which suggests the absence of contaminations by cellular RNA, as shown previously (32, 33) (**Figure 2A**). Principal component analysis (PCA) was performed to determine the overall differences between all miRNAs loaded into EVs and endogenous miRNAs from the cells. The greatest differences were found in miRNA profiles of NHEMs and melanoma cells. The miRNA load of EVs derived from NHEMs and melanoma cells was very similar, but highly different from the corresponding cells (**Figure 2B**). These results were confirmed by correlation analyses, which showed the strongest correlation of miRNA load between EVs derived from melanoma cells and NHEMs (R^2^ = 0.9587) (**Figure 2C**). In contrast, the endogenous (intracellular) miRNA expression of NHEMs and melanoma cells revealed the lowest correlation (R^2^ = 0.6470).

These results indicate a great difference in the expression of endogenous miRNAs between NHEMs and melanoma cells, but a higher similarity of the miRNA load between EVs from NHEMs and melanoma cells.

### Differential miRNA Abundance in EVs and Corresponding Cells

**Figure 3:**
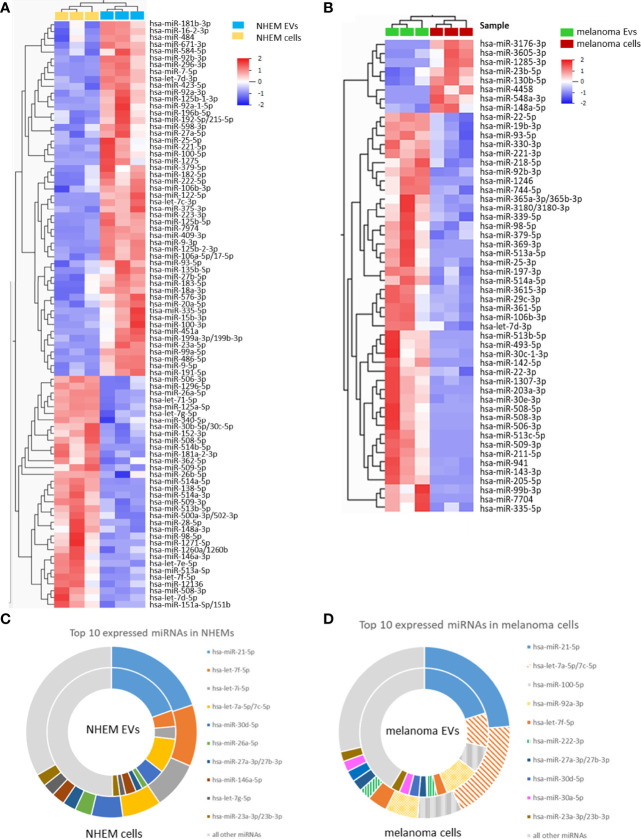
Differential miRNA abundance in EVs and corresponding cells. Heatmaps represent the hierarchical clustering of significantly different miRNAs, comparing NHEMs **(A)** or melanoma cells **(B)** to their corresponding EVs. Pie charts show the absolute quantification (reads per million (RPM)) of the top 10 expressed miRNAs in NHEMs **(C)** or melanoma cells **(D)** compared to their corresponding EVs. Data shown as average of RPMs of three biological replicates.

Comparison between EVs and corresponding cells revealed marked differences of miRNA abundance between EVs and the cells they derived from. For NHEMs, 52 miRNAs were significantly enriched in EVs, while 34 miRNAs showed a higher expression inside the cells (**Figure 3A**, **Table 1**). EVs derived from melanoma cells showed a significant accumulation for 44 miRNAs, while the corresponding melanoma cells showed a higher frequency for eight miRNAs (**Figure 3B**, **Table 2**). When comparing cells and their secreted EVs, six miRNAs (let-7d-3p, miR-106b-3p, miR-335-5p, miR-379-5p, miR-92b-3p, and miR-93-5p) were found to be enriched in EVs from both NHEMs and melanoma cells. In contrast, no miRNAs were found to be more abundant inside NHEMs as well as in melanoma cells.

**Table 1:** Top ten most differential enriched miRNAs in NHEM and NHEM-derived EVs.

miRNA	RPM cells	RPM EVs	log2 fold enrichment	adj. p-value
***enriched in NHEM EVs***				
**hsa-miR-486-5p**	27.73	7920.17	8.16	0.0072
**hsa-miR-223-3p**	1.78	345.59	7.60	0.0051
**hsa-miR-451a**	26.06	3113.14	6.90	0.0333
**hsa-miR-122-5p**	24.25	2310.82	6.57	0.0134
**hsa-miR-335-5p**	8.16	718.49	6.46	0.0366
**hsa-miR-9-3p**	7.55	516.16	6.10	0.0058
**hsa-miR-23a-5p**	14.38	918.05	6.00	0.0186
**hsa-miR-92a-1-5p**	20.84	900.46	5.43	0.0357
**hsa-miR-125b-1-3p**	17.48	661.36	5.24	0.0204
**hsa-miR-296-3p**	14.21	446.87	4.97	0.0124
***enriched in NHEM cells***				
**hsa-miR-513b-5p**	1044.94	221.92	-2.24	0.0121
**hsa-miR-506-3p**	909.44	173.27	-2.39	0.0155
**hsa-miR-513a-5p**	1976.22	351.60	-2.49	0.0277
**hsa-miR-508-5p**	780.43	125.31	-2.64	0.0150
**hsa-miR-1296-5p**	20.71	2.79	-2.89	0.0134
**hsa-miR-508-3p**	7454.42	867.43	-3.10	0.0232
**hsa-miR-514a-3p**	2596.72	273.98	-3.24	0.0146
**hsa-miR-509-5p**	396.49	36.52	-3.44	0.0267
**hsa-miR-514a-5p**	227.58	17.64	-3.69	0.0056
**hsa-miR-146a-3p**	72.94	3.49	-4.38	0.0430

**Table 2:** Top ten most differential enriched miRNAs in melanoma cells and melanoma-derived EVs.

miRNA	RPM cells	RPM EVs	log2 fold enrichment	adj. p-value
***enriched in melanoma EVs***				
**hsa-miR-1246**	12.88	40442.23	11.62	0.0418
**hsa-miR-7704**	7.54	1846.05	7.94	0.0379
**hsa-miR-493-5p**	4.02	292.05	6.18	0.0398
**hsa-miR-369-3p**	13.08	577.75	5.46	0.0349
**hsa-miR-941**	8.27	169.51	4.36	0.0317
**hsa-miR-143-3p**	108.13	1662.16	3.94	0.0353
**hsa-miR-30c-1-3p**	18.19	210.16	3.53	0.0468
**hsa-miR-3615-3p**	18.33	146.52	3.00	0.0393
**hsa-miR-514a-5p**	1.53	11.03	2.85	0.0375
**hsa-miR-29c-3p**	20.32	104.19	2.36	0.0468
***enriched in melanoma cells***				
**hsa-miR-130b-5p**	739.48	391.29	-0.92	0.0342
**hsa-miR-23b-5p**	106.32	26.46	-2.01	0.0405
**hsa-miR-3605-3p**	110.25	27.41	-2.01	0.0413
**hsa-miR-148a-5p**	42.92	7.19	-2.58	0.0406
**hsa-miR-1285-3p**	8.54	n.d.	n.c.	n.c.
**hsa-miR-548a-3p**	15.34	n.d.	n.c.	n.c.
**hsa-miR-3176-3p**	28.49	n.d.	n.c.	n.c.
**hsa-miR-4458**	74.81	n.d.	n.c.	n.c.

n.d., not detected; n.c., not calculated.

The comparison of the absolute values (reads per million (RPM)) of the top 10 expressed miRNAs in NHEMs (**Figure 3C**) or in melanoma cell lines (**Figure 3D**) to the miRNA load of the corresponding EVs revealed large differences in the amount of single miRNAs (**Figures 3C, D**). The top 10 expressed miRNAs represents more than 50% of all detected miRNAs inside of the cells as well as in the EVs (**Figures 3C, D**). However, a large variation was found when comparing miRNAs in cells and in their released EVs. miR-21-5p was found with approximately similar abundance in cells and EVs from NHEMs as well as from melanoma cells. In contrast, compared to corresponding EVs, let-7f-5p and let-7i-5p were highly enriched in NHEM cells, while let-7a-5p showed a 2.5 fold enrichment in melanoma cells.

These different quantitative distributions of miRNAs between cells and EVs thus preclude a stochastic distribution or a mere compartmentalization of cytoplasm, but rather indicate a specific loading mechanism of EV.

### Specific miRNA Enrichment in Melanoma Cell-Derived EVs

**Figure 4:**
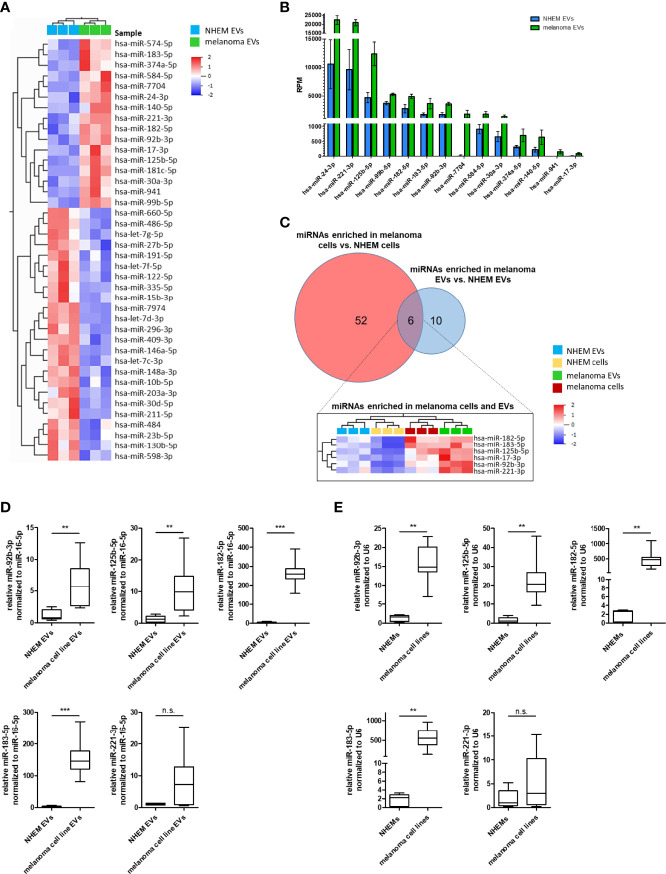
Specific miRNA enrichment in melanoma cell-derived EVs. **(A)** Heatmap presents the hierarchical clustering of significant differential enriched miRNAs in EVs derived from NHEMs and melanoma cells. **(B)** Graph shows the absolute quantification (reads per million (RPM)) of the top enriched miRNAs in melanoma-derived EVs in comparison to NHEM released EVs. **(C)** Venn diagram of miRNAs enriched in melanoma cells and melanoma-derived EVs. The heatmap shows miRNAs enriched in both, melanoma cells and EVs. **(D)** Validation by qRT-PCR of miRNA enrichment in EVs derived from melanoma cell lines and NHEMs. **(E)** Validation of endogenous miRNA expression of NHEMs and melanoma cell lines. Box and whiskers represents median and 5 – 95 percentile of at least five individual NHEM donors and at least two biological replicates from six different melanoma cell lines (A375, WM35, WM9, WM902B, MV3 and BLM) (**p ≤ 0.01; ***p ≤ 0.001; n.s., not significant).

In order to identify melanoma-specific signaling pathways for this type of cell-cell communication, miRNA profiles of EVs derived from NHEMs were compared to those derived from melanoma cells. Overall, most miRNAs in EVs derived from NHEMs and melanoma cells showed a similar distribution. However, by differential enrichment analysis, 16 miRNAs were found to be significantly enriched in melanoma cell-derived EVs, while 24 miRNAs were enriched in EVs released by NHEMs (**Figure 4A**, **Table 3**). The most frequently occurring and significantly enriched miRNAs in EVs from melanoma cells showed a clear increase in absolute quantities (RPM) compared to EVs from melanocytes. Especially miR-24-3p, miR-221-3p and miR-125b-5p were found highly enriched in the melanoma cell-derived EVs (**Figure 4B**), representing 5.6% of all reads, mapped to 490 identified miRNAs.

**Table 3:** miRNAs significantly enriched in melanoma-derived EVs compared to NHEM originated EVs.

miRNA	RPMNHEM EVs	RPMmelanoma EVs	log2 foldenrichment	adj. p-value
**hsa-miR-181c-5p**	n.d.	72.44	n.c.	n.c.
**hsa-miR-7704**	22.46	1846.05	6.36	0.0285
**hsa-miR-941**	8.88	169.51	4.26	0.0340
**hsa-miR-17-3p**	26.68	110.37	2.05	0.0382
**hsa-miR-140-5p**	241.76	652.42	1.43	0.0499
**hsa-miR-125b-5p**	4714.05	12372.22	1.39	0.0280
**hsa-miR-221-3p**	9615.86	20975.48	1.13	0.0271
**hsa-miR-374a-5p**	332.45	711.86	1.10	0.0458
**hsa-miR-24-3p**	10575.16	22418.29	1.08	0.0325
**hsa-miR-183-5p**	1767.06	3678.38	1.06	0.0379
**hsa-miR-92b-3p**	1770.45	3612.54	1.03	0.0141
**hsa-miR-30a-3p**	672.22	1365.21	1.02	0.0380
**hsa-miR-584-5p**	924.33	1794.89	0.96	0.0455
**hsa-miR-182-5p**	2785.11	4923.31	0.82	0.0290
**hsa-miR-99b-5p**	3726.77	5271.35	0.50	0.0135
**hsa-miR-574-5p**	782.71	1078.30	0.46	0.0386

n.d., not detected; n.c., not calculated.

In order to identify a melanoma-specific signature, miRNAs enriched in melanoma cells and melanoma EVs were aligned. Only six miRNAs (miR-17-3p, miR-92b-3p, miR-125b-5p, miR-182-5p, miR-183-5p and miR-221-3p) were identified to be enriched in both melanoma cells and melanoma-derived EVs (**Figure 4C**) and further analysed (e.g. since miR-24-3p was only enriched in melanoma-derived EVs, but not in melanoma cells, it was excluded). Since miR-17-3p was very low expressed, it was excluded from further investigations. To confirm the enrichment for the five remaining miRNAs (miR-92b-3p, miR-125b-5p, miR-182-5p, miR-183-5p and miR-221-3p) in melanoma cell lines as well as in melanoma EVs, qRT-PCRs were performed. Therefore, EVs where isolated from conditioned medium of various melanoma cell lines (A375, WM35, WM9, WM902B, MV3 and BLM) or NHEMs of different donors. A significant enrichment was found for miR-92b-3p, miR-125b-5p, miR-182-5p and miR-183-5p in melanoma cell-derived EVs, as well as in the corresponding cells compared to NHEM-derived EVs and cells (**Figures 4D, E**). For miR-221-3p the enrichment was not significant. These findings mostly correlated with the results of the previous NGS analyses.

### Functional Networks and Biological Pathways of Melanoma Cell and EV Enriched miRNAs

**Figure 5:**
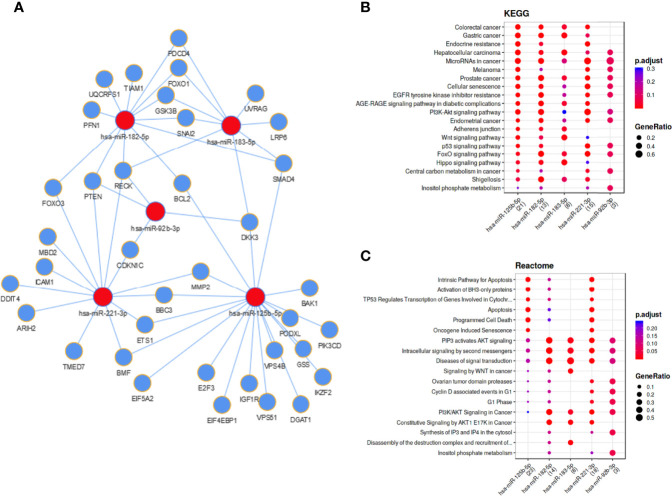
Functional networks and biological pathways of melanoma cell and EV enriched miRNAs. **(A)** Regulatory network based on miRTarBase validated miRNA target prediction. KEGG **(B)** and Reactome **(C)** pathway enrichments for mentioned miRNAs.

For the five investigated miRNAs (miR-92b-3p, miR-125b-5p, miR-182-5p, miR-183-5p and miR-221-3p) an *in silico* network was generated, based on miRTarBase target predictions, using the MIENTURNET (MIcroRNAENrichmentTURnedNETwork) tool (**Figure 5A**). The network included 37 validated target genes for these five miRNAs (**Table 4**). KEGG (Kyoto Encyclopedia of Genes and Genomes) pathway analysis showed strong associations of the identified miRNAs with several cancer entities (e.g. gastrointestinal cancer, hepatocellular carcinoma and melanoma), as well as various signaling pathways (e.g. EGFR, PI3K-AKT, WNT and p53 signaling pathways) (**Figure 5B**). Reactome pathway analysis enriched for apoptosis and cell death associated pathways, oncogene induced senescence and cell cycle associated pathways, as well as signaling pathways including WNT and PI3K/AKT (**Figure 5C**). Similar pathway enhancements were found by KEGG and Reactome analyses using target prediction based on TargetScan, including putative targets (**Figure S2**). The signal pathways found and the possible targets of the miRNA candidates identified here, show the potential of EV-transported miRNAs to influence cells in the tumor microenvironment and thus to promote tumor progression.

**Table 4:** Validated miRNA targets of melanoma cell and EV enriched miRNAs based on miRTarBase.

miRNA-target miRTarBase				
has-miR-125b-5p	hsa-miR-221-3p	hsa-miR-182-5p	hsa-miR-183-5p	hsa-miR-92b-3p
EIF4EBP1	BMF	FOXO3	FOXO1	CDKN1C
BAK1	FOXO3	FOXO1	PDCD4	DKK3
BMF	CDKN1C	PDCD4	LRP6	PTEN
E2F3	TMED7	BCL2	DKK3	RECK
BBC3	DDIT4	PFN1	GSK3B	
BCL2	ARIH2	SNAI2	SNAI2	
ETS1	BBC3	RECK	SMAD4	
DGAT1	ICAM1	SMAD4	RECK	
SMAD4	PTEN	PTEN	UVRAG	
EIF5A2	ETS1	GSK3B		
PIK3CD	RECK	TIAM1		
GSS	MMP2	UQCRFS1		
IKZF2	MBD2			
VPS4B				
VPS51				
MMP2				
IGF1R				
DKK3				
PODXL				

### Enrichment of miR-92b-3p, miR-182-5p and miR-183-5p in EVs Derived From Serum of Melanoma Patients

**Figure 6:**
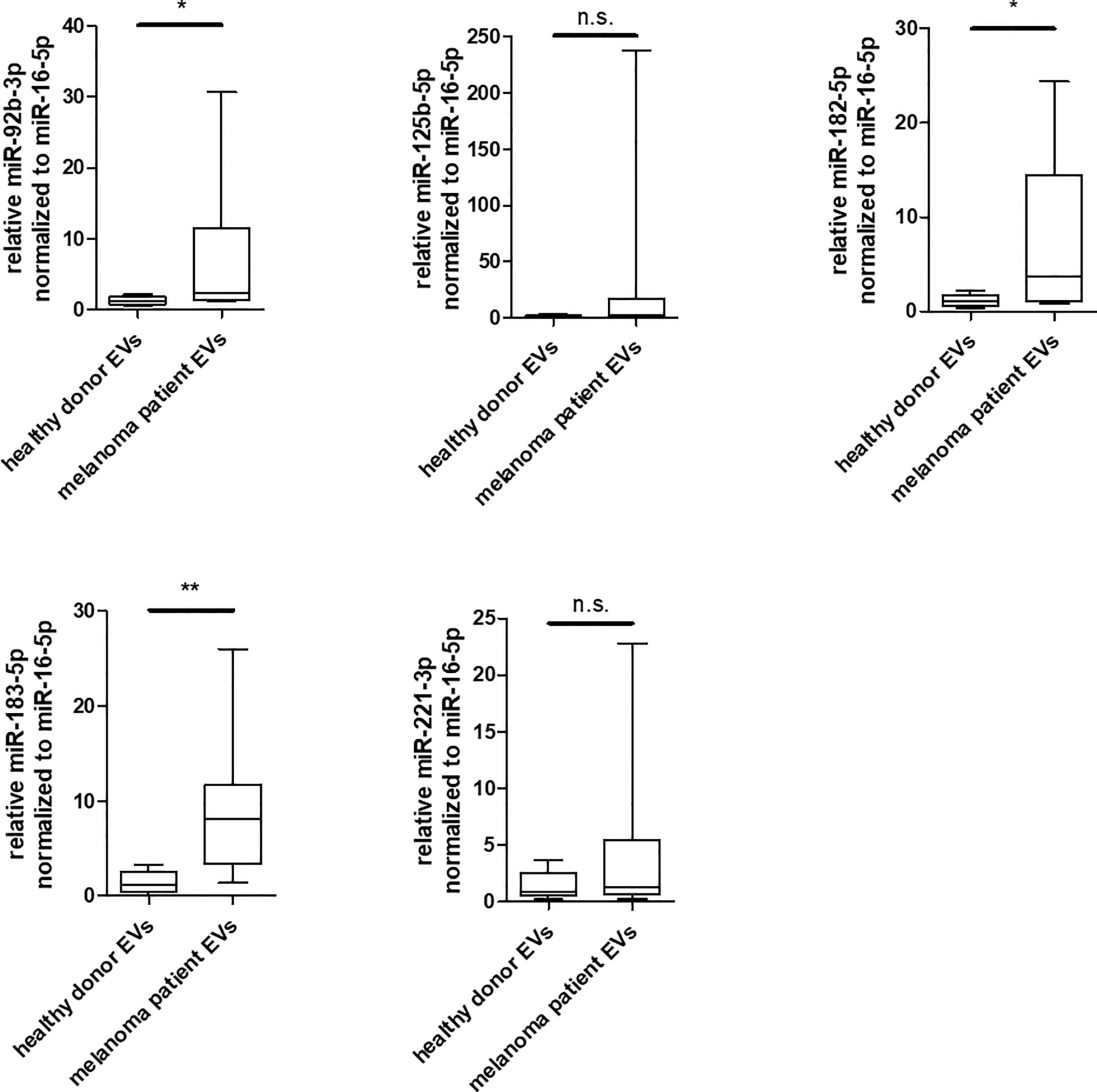
Enrichment of miR-92b-3p, miR-182-5p and miR-183-5p in EVs derived from serum of melanoma patients. Box and whiskers (median and 5 – 95 percentile) represents results of qRT-PCR analyses of indicated miRNAs in EVs derived from serum of individual healthy donors (n = 8) or individual melanoma patients (n = 8) (*p ≤ 0.05; **p ≤ 0.01; n.s., not significant).

To investigate the relevance of the identified miRNAs in melanoma, an initial analysis was performed in a small study cohort (**Supplementary Table 1**). EVs were isolated from serum of 8 patients exclusively with advanced metastatic melanoma and of 8 healthy donors and compared for enrichment of these miRNA by qRT-PCR. In comparison to healthy donors, miR-182-5p and miR-183-5p and to some extent also miR-92b-3p were found to be significantly enriched in EVs derived from melanoma patients (**Figure 6**), while miR-125b-5p and miR-221-3p showed no significant differences between healthy donors and melanoma patients. Especially miR-183-5p was clearly increased and showed an average enrichment of 5.9 fold compared to the healthy donors. These results agree with the previous *in vitro* analyses of this study.

## Discussion

The characterization of isolated EVs derived from normal melanocytes and melanoma cell lines showed high similarities for marker expression and size distribution, while the amount of released EVs was increased for the melanoma cell lines compared to melanocytes. This difference was most prominent for the metastatic cell line WM9. Previous studies revealed that secretion of EVs by several cancer cells is enhanced compared to normal cells (34, 35). A possible reason for higher release of EVs by malignant cells could be an increased expression and activity of regulators of EV secretion. As such this has been shown in some cancers for e.g. ESCRT components, syntenin, heparanase, small GTPases (such as Rab27A and Rab27B), SNARE proteins (such as SNAP23) (36–39).

By next generation sequencing analysis we revealed that melanoma cell lines and melanocytes showed a very different endogenous miRNA expression profile. miRNA load showed a stronger correlation between EVs derived from melanocytes and EVs from melanoma cells, than EVs with their corresponding cells. The enrichment of certain miRNAs in EVs compared to cytosol indicates that loading of EVs with miRNAs is not just a stochastic distribution, but must be the result of actively regulated processes. This is further confirmed when comparing those miRNAs which are most highly expressed in the cells with their concentration in corresponding EVs (e.g. miRNA let-7a-5p was highly expressed in melanoma cells, but only lowly accumulated in EVs of these cells) (**Figure 3C, D**). Similar results have also been reported in canine melanoma, showing different frequencies of individual miRNAs in cells and EVs (40).

Some mechanisms for selective loading of miRNAs to EVs have recently been suggested. As such, RNA binding proteins [e.g. A2B1 (41, 42), Ago2 (43), YBX1 (44, 45), MEX3C (46), MVP (47), HNRNPA1 (48) or SYNCRIP (49)] were shown to regulate sorting of specific miRNAs into EVs. Besides, membrane proteins CAV1 (50), nSMASE2 (51) and VPS4A (52) contribute to miRNA loading into EVs. In addition, the autophagy associated LC3-conjugation machinery mediates miRNA abundance in EVs, by specific loading of RNA-binding proteins into EVs (53). Several factors involved in the loading machinery of EVs have already been described, but more research needs to be done to understand the specific selection of components for individual EVs.

Assuming that miRNAs are selectively loaded into EVs, the miRNA cargo in EVs derived from melanocytes and melanoma cells was compared to identify melanoma specific enrichment of miRNAs. 24 miRNAs were significantly enriched in melanocyte-derived EVs, while in EVs from melanoma cells 16 miRNAs were increased.

These results are consistent with a previous report on malignant melanoma, comparing miRNA profiles in exosomes from HEMa-LP and A375 cell line (54). It identified nine miRNAs (miR-574-5p, miR-584-5p, miR-140-5p, miR-221-3p, miR-182-5p, miR-92b-3p, miR-17-3p, miR-125b-5p and miR-30a-3p) to be enriched in exosomes derived from A375, these findings being consistent with the significantly accumulated miRNAs in EVs from melanoma cells in our report. The limitation of that study was the comparison of melanocytes from only one donor with only one cell line (A375). In contrast, we compared three different melanoma cell lines representing melanoma in different progression stages with human primary melanocytes from three different donors, which better eliminates inter-individual differences.

In order to find general melanoma-specific miRNAs that are enriched in EVs of most malignant melanomas, miRNAs were searched that are more strongly expressed in melanoma cells and more enriched in melanoma EVs than in melanocytes and their EVs. This approach identified six miRNAs (miR-17-3p, miR-92b-3p, miR-125b-5p, miR-221-3p, miR-183-5p and miR-182-5p). Because miR-17-3p had very low expression values, it was excluded for further analysis. For the miRNAs: miR-92b-3p, miR-125b-5p, miR-183-5p and miR-182-5p significant higher levels in melanoma cells and their derived EVs could be validated by qRT-PCR (**Figure 4**). These results confirm our NGS data, although the differences in the qRT-PCRs appear to be stronger, which could be explained by different normalization methods and additionally included melanoma cell lines. Our findings also agree with a study, showing that miR-182-5p as well as miR-183-5p was strongly upregulated in cells originated from primary melanoma tumors compared to normal melanocytes (55).

One of the most abundant miRNAs in EVs derived from melanoma cells was miR-24-3p. Although it was found to be two-fold enriched in melanoma cell-derived EVs compared to EVs released from NHEMs, it was not expressed at a higher level in melanoma cells compared to NHEMs. For this reason, we excluded this miRNA from further analyses in this study. Patients with oral squamous cell carcinoma also showed accumulation of miR-24-3p in salivary exosomes compared to healthy donors. In contrast to our study, oral squamous cell carcinoma tissue also showed an enrichment for miR-24-3p compared to peritumoral tissue (56). Furthermore, the enrichment of miR-24-3p could be found in EVs derived from nasopharyngeal carcinoma cell lines and patient sera (57), head and neck cancer cell lines (58) and classical Hodgkin’s lymphoma (59).

Functional analyses of miR-24-3p in the mouse melanoma cell line B16F10 showed that it acts as a tumor suppressor by inhibiting migration, invasion and proliferation by directly targeting p130Cas (60).

KEGG and Reactome pathway analysis for the miRNAs that met our selection criteria showed associations with different cancer entities and cancer signaling pathways (**Figure 5**).

For these *in silico* analyses of functional networks and biological pathways miRTarBase was used because it accesses experimentally validated miRNA targets. This procedure offers a more precise generation of the biological function of the respective miRNAs than the use of TargetScan, a procedure based on putative target prediction by sequence complementarity.

When comparing our results with data obtained from other malignant tumors, one finds that the miRNAs identified here, are also enriched in EVs derived from cancer cells of other entities (**Table 5**). These results further indicate that the miRNAs contained in EVs could present liquid biopsy markers in different cancer entities.

**Table 5:** **miRNAs** significantly enriched in melanoma cells and EVs are also enriched in EVs derived from other cancer entities.

EV enriched miRNA	cancer entity	references
**hsa-miR-17-3p**	ovarian cancer cells lung cancer patients	(61) (62, 63)
**hsa-miR-92b-3p**	lung cancer patients synovial carcinoma cell lines and patients gastric cancer patients	(16) (21) (64)
**hsa-miR-125b-5p**	melanoma cells prostate cancer cell lines chemoresistant diffuse large B-cell lymphoma patients	(9, 54, 65, 66) (67) (68)
**hsa-miR-182-5p**	breast cancer cells glioblastoma patients	(69) (70)
**hsa-miR-183-5p**	glioma patients colorectal cancer cells prostate cancer cells melanoma patients	(20, 70) (71) (67) (72)
**hsa-miR-221-3p**	cervical carcinoma cells colorectal cancer cells oral squamous cell carcinoma cells	(17, 73, 74) (75) (76)

With regard to predicted or proven functions and their potential in the tumor microenvironment, the six miRNAs identified here, were mainly linked to angiogenesis when transported by cancer cell-derived EVs. Several functional studies in different entities have shown that miR-183-5p, miR-182-5p, miR-221-3p and the miR-17-92 cluster induce angiogenesis when taken up by epithelial or endothelial cells. This was achieved through the inhibition of factors as FOXO1 (71), CMTM7 (69), VASH1 (17), THBS2 (73), SOCS3 (75), PIK3R1 (76), TSP-1 and CTGF (77).

Some of the identified miRNAs in addition have the potential to mediate the function of tumor associated immune cells, either stimulating the inflammatory response or suppressing them. We reported earlier that miR-125b-5p delivered by melanoma cell line-derived EVs into macrophages induces a pro-inflammatory phenotype by targeting LIPA (9). In addition, it was shown that in γδ T-cells miR-125b-5p mediates downregulation of activation and cytotoxicity (78). Further, miR-221-3p drives M2 macrophages to a pro-inflammatory function by directly targeting JAK3 in rheumatoid arthritis (79). Similarly, exosomal miR-183-5p derived from mouse breast cancer cells induces a pro-inflammatory cytokine profile in macrophages, contributing to tumor progression in a mouse model (80).

Taken together, these results demonstrate the potency of miRNAs transported by cancer cell-derived EVs to mediate a variety of cells (e.g. macrophages) within the tumor microenvironment to drive tumor progression.

Besides their biological functions, there is consensus that some miRNAs transported by EVs, can be used as diagnostic or prognostic liquid biopsy markers for melanoma (81). We therefore performed qRT-PCR analyses for the previous validated miRNAs and found the miR-92b-3p, miR-182-5p and miR-183-5p to be significantly enriched in EVs derived from melanoma patients in comparison to healthy donors (**Figure 6**). These miRNAs have not been yet described as potential liquid biopsy markers in melanoma. It is noteworthy that in our cohort, even despite the small sample size, miR-183-5p were higher in each patient than in any control. Due to the limitations in the cohort of patients analyzed, such as the age differences in the groups and the small number of samples, the results obtained are only to be seen as an indication and should therefore be validated in an additional cohort. To our knowledge, miR-182-5p and miR-183-5p have only been reported as potential exosomal biomarkers in glioma (20, 70), while exosome embedded miR-92b-3p is described as biomarker in synovial sarcoma (21) and gastric cancer (64).

So far, the number of studies on EV embedded miRNAs of melanomas as biomarkers is limited. miR-1180-3p was reported as potential exosomal diagnostic marker, but in contrast to the miRNAs reported here, this miRNA was shown to be reduced in melanoma patients (72). The miR-222 was proposed as a diagnostic marker in melanoma, but it has not been analysed in patient-derived EVs, but only in cell lines derived from patients (82). The miR-17, miR-19a, miR-21, miR-126, and miR-149 were found to be enriched in plasma-derived exosomes from melanoma patients (83), but since were isolated directly from plasma by ExoQuick reagent without excluding larger vesicles or free plasma circulating RNAs, the detected concentrations of miRNAs may not all reproducibly originate only from EV as defined in our study.

Our results support the hypothesis that miRNAs derived from EVs can serve as prognostic or diagnostic liquid biopsy markers in melanoma. We identified EV-derived miRNAs and showed that those miRNAs, which were enriched in melanoma cells and EVs, are also found elevated in serum-derived EVs of patients with metastatic melanoma, but not in healthy subjects.

Its verification by clinical trials is beyond the scope of this study, but provides a worthwhile outlook.

For miR-125b-5p a significant enrichment could only be shown in EVs derived from melanoma cell lines, but not for melanoma patients. A possible explanation for these contrasting results could be, that the patients analysed here, mostly had an advanced melanoma, while miR-125b-5p accumulation in EVs was reported to be reduced in advanced melanoma stages (65).

Albeit, high levels of serum circulating miR-221-3p were examined as new prognostic marker in melanoma patients (84), here no different enrichment of miR-221-3p could be found in EVs of melanoma patients and healthy donors. Taken together, this indicates that there are strong differences in the significance of potential biomarkers depending on origin.

The here identified miRNAs enriched in melanoma EVs as well as in melanoma cells, are functionally associated with different tumor entities and signaling pathways involved in cancer progression. Furthermore, three miRNAs (miR-92b-3p, miR-182-5p and miR-183-5p) could be confirmed enriched in EVs derived from serum of melanoma patients. Taken together, the accumulation of cancer associated miRNAs in melanoma-derived EVs, even in patients, emphasizes their potential as novel prognostic or diagnostic liquid biopsy markers.

## Data Availability Statement

The datasets presented in this study can be found in online repositories. The names of the repository/repositories and accession number(s) can be found here: https://data.mendeley.com/datasets/g9b8k73vzx/2, DOI: 10.17632/g9b8k73vzx.2.

## Ethics Statement

The studies involving human participants were reviewed and approved by Ethical committee of the medical faculty of the Martin-Luther-University Halle-Wittenberg. The patients/participants provided their written informed consent to participate in this study.

## Author Contributions

Conceptualization, DG and SK-H; Data curation, DG and SK-H; Formal analysis, DG; TEM, GH; Investigation, DG and TK; Supervision, DG and CS; Collection of patient sample JE, LG; Visualization, DG; Writing – original draft, DG, SK-H and TK; Writing – review & editing, DG, SK-H and CS. All authors contributed to the article and approved the submitted version.

## Funding

This study was supported by Wilhelm Roux Funding Program (FKZ 31/43) by the medical faculty of the Martin-Luther-University Halle-Wittenberg. We acknowledge the financial support within the funding program Open Access Publishing by the German Research Foundation (DFG).

## Conflict of Interest

The authors declare that the research was conducted in the absence of any commercial or financial relationships that could be construed as a potential conflict of interest.

## Publisher’s Note

All claims expressed in this article are solely those of the authors and do not necessarily represent those of their affiliated organizations, or those of the publisher, the editors and the reviewers. Any product that may be evaluated in this article, or claim that may be made by its manufacturer, is not guaranteed or endorsed by the publisher.
