# Supplementary figures and images for: Comprehensive Analyses of miRNAs Revealed miR-92b-3p, miR-182-5p and miR-183-5p as Potential Novel Biomarkers in Melanoma-Derived Extracellular Vesicles
Source: Front Oncol. 2022 Jul 8;12:935816. doi: 10.3389/fonc.2022.935816 (PMC9309285; doi:10.3389/fonc.2022.935816)

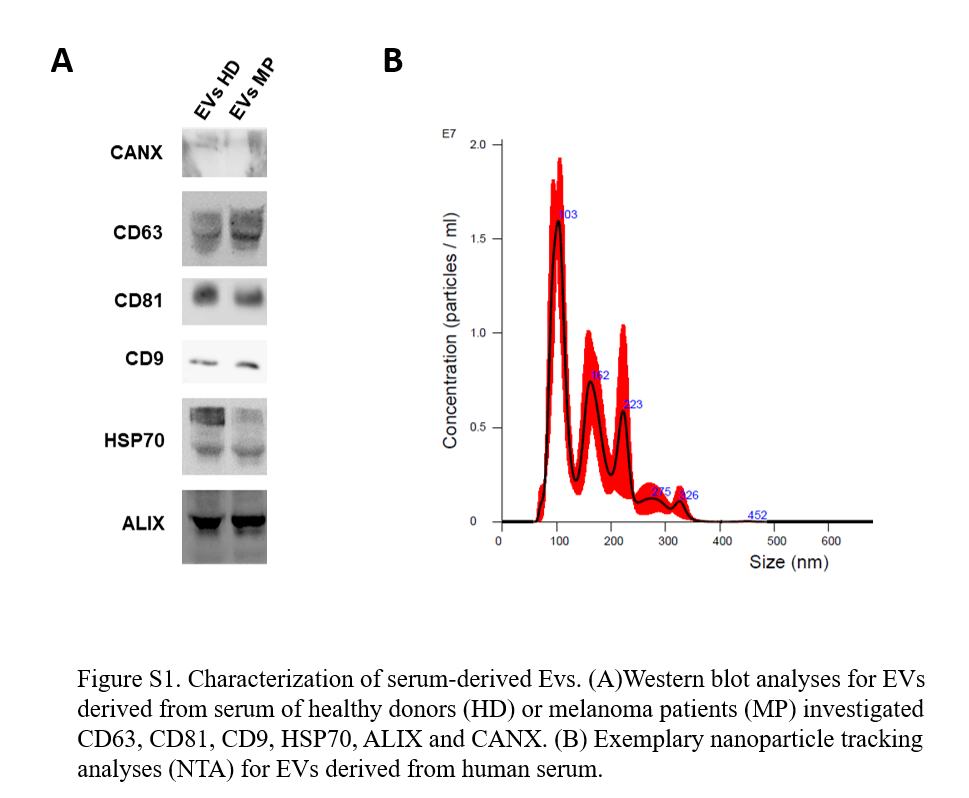

Supplement: Supplementary file 1 [file Image_1.tif]

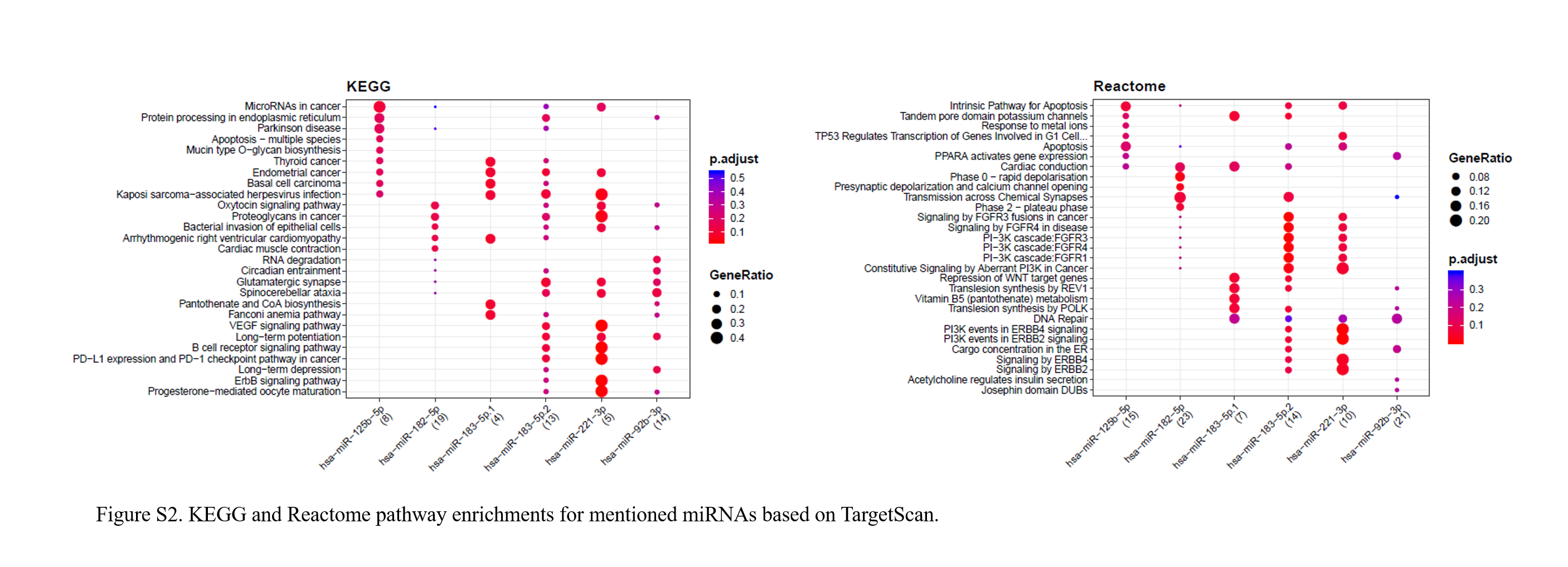

Supplement: Supplementary file 2 [file Image_2.tif]
