# Supplementary material for: Comprehensive Analyses of miRNAs Revealed miR-92b-3p, miR-182-5p and miR-183-5p as Potential Novel Biomarkers in Melanoma-Derived Extracellular Vesicles
Source: Front Oncol. 2022 Jul 8;12:935816. doi: 10.3389/fonc.2022.935816 (PMC9309285; doi:10.3389/fonc.2022.935816)
Supplement: Supplementary file 3 [file Table_1.docx]

**Supplemental Table1.** Characteristics of patients and healthy donors

| **Total** | **Healthy Volunteer** | **Melanoma** |
| --- | --- | --- |
| **n = 18** | **n = 8** | **n = 8** |
| **Age, years** | 35 | 71 |
|  | (24-41) | (54-80) |
| **Gender** |  |  |
| Male | 2 | 3 |
| Female | 6 | 5 |
|  |  |  |
| **Melanoma Stage** |  |  |
| IIIC  IV | N/A  N/A | 1  7 |
